# Real Time Identification of Drug-Induced Liver Injury (DILI) through Daily Screening of ALT Results: A Prospective Pilot Cohort Study

**DOI:** 10.1371/journal.pone.0042418

**Published:** 2012-08-14

**Authors:** Helmi M'Kada, Hugo Perazzo, Mona Munteanu, Yen Ngo, Nittia Ramanujam, Bruno Fautrel, Françoise Imbert-Bismut, Vlad Ratziu, Ina Schuppe-Koistinen, Véronique Leblond, Jean Yves Delattre, Yves Samson, Olivier Lyon Caen, François Bricaire, David Khayat, Charles Pierrot-Deseilligny, Serge Herson, Zahir Amoura, Patrick Tilleul, Olivier Deckmyn, Pierre Coriat, Vincent Nicolas Delpech, Philippe Boulogne, Dominique Bonnefont-Rousselot, Thierry Poynard, for the Drug Induced Liver Injury Groupe Hospitalier Pitié-Salpêtrière (DILI-GHPS) Group and the Safer and Faster Evidence-based Translation (SAFE-T) Consortium

**Affiliations:** 1 Service de Biochimie Métabolique, Groupe Hospitalier Pitié-Salpêtrière (GHPS), Assistance Publique Hôpitaux de Paris (AP-HP), Paris, France; 2 AP-HP, University Pierre Marie Curie (UPMC) Liver Center, Paris, France; 3 Biopredictive, Paris, France; 4 Rheumatology Unit, GHPS, Paris, France; 5 Astra Zeneca, Södertälje, Sweden; 6 Hematology Unit, GHPS, Paris, France; 7 Neurology-2 Unit, GHPS, Paris, France; 8 Cerebral Vascular Emergency Unit, GHPS, Paris, France; 9 Neurology-4 GHPS, Paris, France; 10 Infectious Disease Unit GHPS, Paris, France; 11 Oncology Unit GHPS, Paris, France; 12 Neurology-1 GHPS, Paris, France; 13 Internal Medicine-2 GHPS, Paris, France; 14 Internal Medicine-1 GHPS, Paris, France; 15 Pharmacology Unit, GHPS, Paris, France; 16 Intensive Care Anesthesiology GHPS, Paris, France; 17 Administrative Direction GHPS, Paris, France; 18 Information Technology Unit GHPS, Paris, France; 19 EA 4466, Faculté des Sciences Pharmaceutiques et Biologiques, Université Paris Descartes, Sorbonne Paris Cité, Paris, France; Duke University, United States of America

## Abstract

**Objective:**

Identification of drug-induced liver disease (DILI) is difficult, even among hospitalized patients. The aim of this pilot study was to assess the impact of a specific strategy for DILI screening.

**Design:**

We prospectively compared the number of acute DILI cases identified in one week of a proactive strategy based on centralized elevated ALT values to those identified with a standard of care strategy for 24-week period based on referral cases to the hepatology unit. In the centralized strategy, a designated study biochemist identified patients with ALT greater than 3 times the upper limit of normal values (ULN) and notified the designated hepatologists, who then went to the patients' wards, analyzed the charts, and if necessary, interviewed the identified patients. During these two periods, patients with possible DILI were included after signing an informed consent in an ongoing European diagnostic study (SAFE-T consortium).

**Results:**

During the 24-week period of the standard strategy, 12 (0.04%) patients out of a total of 28,145 were identified as having possible DILI, and 11 of these accepted to be included in the protocol. During the one-week proactive period, 7 patients out of a total of 1407 inpatients (0.498%) [odds ratio vs. standard = 12.1 (95% CI, 3.9–32.3); P<0.0001] were identified with possible DILI, and 5 were included in the protocol.

**Conclusion:**

A simple strategy based on the daily analysis of cases with ALT >3 ULN by designated biochemists and hepatologists identified 12 times more acute cases of drug-induced liver disease than the standard strategy.

This pilot cohort is registered on the number AP-HP P110201/1/08-03-2011 and AFSSAPS B110346-70.

## Introduction

Adverse drug events occur as a consequence of medication errors or adverse drug reactions (ADR). ADRs rate among the leading causes of death in the Western world [1]–[2]. Systematic reviews have shown that 5–10% of all inpatients are expected to experience severe ADRs, which have contributed to the deaths of 0.3–0.5% of all admitted patients [1]–[2]. However, 30–40% of those ADR are considered to have been preventable.

Drug-induced liver injury (DILI) due to paracetamol overdose and idiosyncratic drug reactions is the leading cause of acute liver failure and may contribute to as many as 0.3% of all inpatient deaths [3]–[5].

Approximately 1 in 100 patients develops DILI during hospitalization, but more than 50% of cases have been missed when they occurred in non-hepatology departments [4]. Therefore better methods of DILI detection in hospitals are needed.

The Safer and Faster Evidence-based Translation (SAFE-T) consortium is a public–private partnership comprising 20 partners from the pharmaceutical industry, small–medium enterprises, academic institutions and clinical units of excellence, with representatives from the European Medicines Agency (EMA) as external observers and advisors. It operates under the framework of the EU Innovative Medicines Initiative Joint Undertaking (IMI-JU) (http://www.imi.europa.eu) [5]–[8].

As a part of the protocols initiated by SAFE-T, we aimed to improve the detection of DILI in hospitals by organizing a centralized alert algorithm. This strategy was based on the standard “Temple's criteria", which is the occurrence of an elevated alanine aminotransferase serum level (ALT) greater than 3 times the upper limit of normal (ULN) in the presence of a drug and in the absence of other causes [9]–[11].

We report here on the results of a pilot study describing the proof of concept of a proactive strategy based on a daily centralized analysis of elevated ALT (centralized strategy) compared to the usual “passive" strategy based on cases referred to the hepatology unit (standard strategy). The centralized strategy identified 12 times more patients with DILI than the standard strategy.

## Patients and Methods

This study was part of the ongoing protocol (Protocol 3 of the Work Package 3) of the SAFE-T consortium, which was in accordance with the Helsinki Declaration and approved by the Pitié Salpêtrière Hospital Ethics Committee. All included patients signed an informed consent. The protocol for this cohort and supporting STROBE checklist are available as supporting information; see Protocol S1 and Checklist S1.

The SAFE-T consortium proposes a generic qualification strategy for translational safety biomarkers (TSBM), outlining proposals on how to generate sufficient preclinical and clinical evidence to qualify new TSBM for regulatory decision-making in defined contexts. The experience gained during the course of the SAFE-T project for three organ toxicities will be integrated into improvements for this initial generic approach [5]–[8].

### Centralized strategy

The “SAFE-T group" of the study included 8 participants. All ALT assay results performed in the two laboratories of the hospital were centralized each day by a senior biochemist (HMK) and a technician (NR). The results of all elevated ALT samples greater than 3×ULN (ALT>3ULN) were sent by intranet to 6 experienced hepatologists (HP, YN, MM, MR, JM, and TP). In the 48 hours following the ALT results, one of the hepatologists went to the inpatient wards and analyzed the chart. According to the patient's chart, each case with ALT>3ULN was classified as: hepato-biliary disease (such as viral hepatitis C or cholelithiasis), cardiovascular origin (such as septic shock or cardiac arrest), muscle disease (such as contusion, myopathy), other causes, or possible DILI according to the SAFE-T protocol definition [6]–[8]. The criteria for inclusion as acute DILI in the SAFE-T protocol were the following: consecutive patients with suspected acute DILI, defined as ALT>3ULN, and with baseline results before drug treatment, if available, below the ULN; history of drug intake including acetaminophen, multiple drugs, recreational drugs (cocaine, ecstasy, amphetamines), and herbs; a minimum 2-fold increase from the baseline level if the baseline ALT level, if available, was greater than ULN; absence of other known causes of liver injury; and patients aged >18 years who were capable of and willing to provide written informed consent. The exclusion criteria were the following: hepatitis E, hepatitis C, autoimmune liver disease, primary biliary cirrhosis, primary sclerosing cholangitis, extrahepatic cholestasis, ischemic liver damage and the presence of liver metastasis or other malignant diseases. If the patient met the inclusion criteria, and the medical team agreed, the hepatologist interviewed the patient, explained the protocol and included the patient in the SAFE-T protocol after the consent was signed.

Patients with DILI had liver injury classification based on the first laboratory determination according to the CIOMS (Council for International Organizations of Medical Science) criteria [12].

### Standard strategy

The standard strategy was the routine followed in the hospital. The suspected DILI cases were presented by the non-hepatologist physician to a senior full-time hepatologist; these were then considered for inclusion in the SAFE-T protocol if they met the inclusion criteria, as with the centralized strategy.

### Biochemical analysis

Biochemical assays were performed with fresh plasma that had been decanted and stored for a maximum of 72 hours at +2 to 8°C under no-light conditions. ALT-activity measurement used the reference method defined by the International Federation of Clinical Chemistry and Laboratory Medicine (IFCC) with pyridoxal phosphate and was calibrated [11]. Total bilirubin was assayed by the diazo reaction method.

The ALT ULN was based on a study of 2,200 apparently healthy blood donors whowere negative for human immunodeficiency virus, hepatitis B virus, and hepatitis C virus markers. The study included 1,171 men and 880 women. The thresholds were 26 IU/L in women and 35 IU/L in men. They were determined by the mean +1 SD after exclusion of the 5% extreme values [7], [11].

### Statistical analysis

The main endpoint was the prevalence of possible acute cases of DILI identified during the period. The a priori hypothesis was that a centralized strategy would result in a minimum 6-fold increase in the prevalence of identified DILI cases, i.e. from one DILI every two weeks, which was the standard incidence we observed, to three DILI every week. The prevalences were compared using odds ratios and Z-tests, and patient characteristics were compared with the Fisher's exact test and Mann-Whitney test.

## Results

### Centralized period

**Figure 1:**
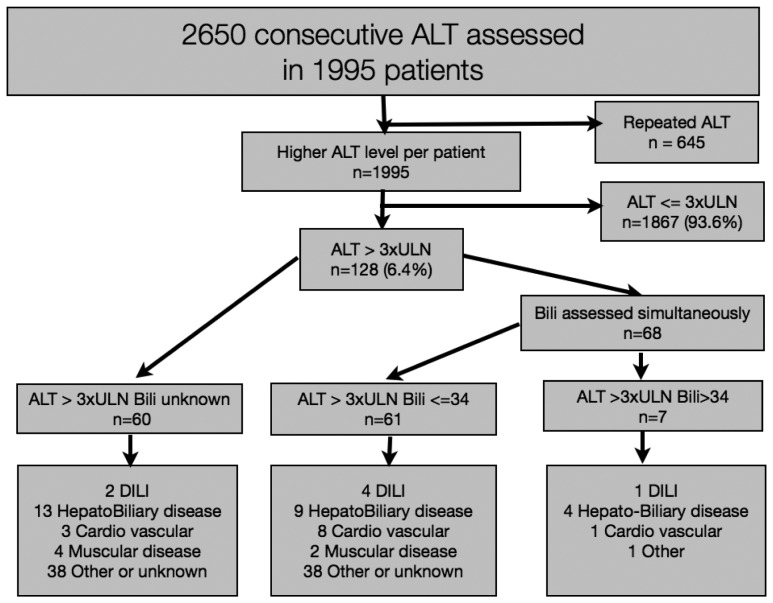
Flowchart of the identification of DILI during the centralized period in 1995 patients with ALT measurement.

During the one-week pro-active period, from November 26 to December 2 2011, 1,995 patients had ALT measurements (Figure 1), including 1,407 inpatients and 588 outpatients. The characteristics of patients with and without elevated ALT during the centralized period are described in Table 1. A total of 128 patients had ALT >3ULN. Most cases of elevated ALT were identified in patients who were hospitalized in the intensive care, hepatology and neurology units.

**Table 1:** Characteristics of patients analyzed during the centralized period.

Characteristic	ALT >3ULN	ALT ≤3ULN
	n (% prevalence)	n (% 1-prevalence)
**Number of patients (% of 1995)**	**128 (6.4)**	**1867 (93.6)**
Gender		
Male n, (%)	66 (6.0)	1033 (94.0)
Female	62 (6.9)	834 (93.1)
Medical Unit inpatients	104 (7.4)	1303 (92.6)
Intensive Care	30 (15.8)	160 (84.2)
Hepatology	14 (10.5)	133 (90.5)
Neurology	14 (9.3)	136 (90.7)
Liver/Biliary surgery	9 (16.7)	45 (83.3)
Internal medicine	8 (5.0)	151 (95.0)
Hematology/Oncology	7 (8.0)	80 (92.0)
Infectious disease	3 (4.4)	65 (95.6)
Rheumatology	4 (7.7)	48 (92.3)
Pulmonology	4 (12.9)	27 (87.1)
Cardiology	1 (1.8)	55 (98.2)
Psychiatry	1 (7.1)	13 (92.9)
Other inpatients	15 (2.8)	364 (97.2)
Outpatients	24 (4.1)	564 (95.9)
ALT (IU/L) median, (range)		
Male	155 (108–925)	28 (5–105)
Female	124 (79–1485)	21 (4–78)
Total bilirubin (micromol/L)		
Male	17 (3–164) n = 35	8 (3–600) n = 378
Female	7 (3–583) n = 33	6 (3–209) n = 301
Age (years)	53.6 (18.5–89.2)	57.2 (2.2–101.5)

The characteristics of patients with and without DILI are given in Table 2. Seven DILI cases were identified; these occurred only among inpatients, with a prevalence of 6.7% (7/104). These patients were hospitalized in the intensive care, neurology, hepatology, infectious disease and psychiatric units. Five of the seven patients accepted inclusion in the SAFE-T protocol.

**Table 2:** Characteristics of patients with possible DILI among patients with ALT >3ULN during the centralized period.

	DILI	No DILI
Characteristics	n (% prevalence)	n (% 1-prevalence)
**Number of patients (% of 128)**	**7 (5.5)**	**121 (94.5)**
Gender		
Male	4 (6.1)	62 (93.9)
Female	3 (4.8)	59 (95.2)
Patients status		
Outpatients	0 (0)	24 (100)
Inpatients	7 (6.7)	97 (93.3)
Intensive Care	2 (6.7)	28 (93.3)
Neurology	2 (14.3)	12 (85.7)
Hepatology	1 (7.1)	13 (92.9)
Infectious disease	1 (33.3)	2 (66.7)
Psychiatry	1 (100)	0 (0)
Liver/Biliary surgery	0 (0)	9 (100)
Internal medicine	0 (0)	8 (100)
Hematology/Oncology	0 (0)	7 (100)
Rheumatology	0 (0)	4 (100)
Pulmonology	0 (0)	4 (100)
Cardiology	0 (0)	1 (100)
Other inpatients	0 (0)	7 (100)
Age (years) median, (range)	54.2 (23.1–78.3)	53.1 (18.5–89.2)
ALT (IU/L) median, (range)	221 (140–1485)	142 (79–1140)
Total bilirubin (micromol/L)	12 (6–39)	10 (3–583)
ALT >3ULN during hospitalization	1	0
ALT >3ULN on admission	6	0

### Standard period

During the 24-week period of the standard strategy (June 1-November 25, 2011), 12 patients out of a total of 36,077 inpatients were identified with possible DILI, i.e. a prevalence of 0.033%; 11 of these accepted inclusion in the SAFE-T protocol.

### Main endpoint

During the one-week centralized period, the prevalence of DILI was 0.498% among inpatients, (7 patients out of a total of 1,407 inpatients) which was significantly higher than the prevalence observed during the standard period, 0.043% (12/28,145 inpatients), with an odds ratio vs. standard of 12.1 (95% CI, 3.9–32.3; P<0.0001).

### Comparison between the two periods

The two periods had the following similar characteristics (Table 3, Table 4 and Table S1): number of inpatients and outpatients per week, the percentage of patients who underwent ALT assays, and the percentage of patients with ALT >3ULN. All patients period had ALT elevation after history of drug intake. In the group of patients who had multiple suspected drugs, 87% (13/15) started all the medications at the same time. The individual characteristics of DILI patients identified during the centralized period were less severe than those identified during the standard period, with significantly lower ALT (median 221 vs. 1,498 IU/L, P<0.001); though statistically non-significant, there were also fewer patients with jaundice (Hy's rule criteria: 1 vs. 5; P = 0.20) and no deaths or transplantations (0 vs. 4; P = 0.30) (Table 4).

**Table 3:** Characteristics of patients with DILI according to the screening period.

Characteristics	Period Strategy	
	Centralized	Standard
Duration of screening (2011)	1 week: Nov 26–Dec 2	24 weeks: June 1–Nov 25
Total patients	12 895	179 538
Inpatients	3 349	36 077
Outpatients	9 546	143 461
Patients with ALT measured	1 995	30 977
Inpatients	1 407	28 145
Outpatients	588	2 832
Number of patients with ALT >3ULN (%)	128 (6.4%)	2 685 (4.9%)
Inpatients	104	1 226
Outpatients	24	1 459
**Inpatients with possible DILI**	**7**	**12**
Male/Female	4/3	9/3
Age (years) median, (range)	54 (18–77)	55 (22–63)
Outpatients/Inpatients	0/7	0/12
Hepatology/Other ward	1/6	12/0*
ALT, IU/L median, (range)	221 (140–1485)	1498 (215–15896)*
Total bilirubin, micromol/L	12 (6–39)	29 (6–491)
Hy's criteria, yes/no	1/6	5/7
Death/transplantation/none	0/0/7	3/1/8
RUCAM score		
Possible/probable/highly probable	0/4/3	4/2/6
DILI type hepatocellular/mixed	6/1	11/1
Included yes/no SAFE-T DILI protocol 3	5/2	11/1

* P<0.001 Hy's criteria ALT >3ULN and total bilirubin >31 micromol/L.

**Table 4:** Characteristics of patients identified with acute DILI.

#	Gender/Age	Liver injury	RUCAM score (class)	SAFE-T	Outcome (Death)	Drugs	Indication of drug
**Centralized period**							
1	M/56	HC	9 (highly probable)^1^	yes	no	methylprednisolone; methotrexate; leucovorin	cerebral lymphoma
2	M/45	HC	7 (probable)^1^	yes	no	acetaminophen (3 g) rifampicin; thiamphenicol	vertebral surgery sepsis
3	F/77	HC	9 (highly probable)^1^	yes	no	vincistine; methotrexate; leucovorin	cerebral lymphoma
4	F/53	HC	7 (probable)^1^	yes	no	pyrimethamine; sulfadiazine; leucovorin	cerebral toxoplasmosis AIDS
5	M/18	HC	8 (probable)	yes	no	olanzapine	acute delirium
6	F/53	HC	9 (highly probable)^1^	no	no	fludarabine, busulfan, antilymphocyte serum	bone marrow transplantation
7	M/27	Mixed	6 (probable)	no	no	acetaminophen (2 g); tramadol	craniocerebral trauma
**Standard period**							
1	M/61	HC	7 (probable)^1^	yes	no	rituximab; fludarabine	leukemia
2	F/22	HC	5 (possible)^1^	yes	yes	carbamazepine; trimethoprim sulphamethoxazole; prednisone; valacyclovir; sirolimus	lymphoproliferative disorder
3	M/54	HC	10 (highly probable)	yes	no	acetaminophen (8 g)	abdominal pain
4	F/31	HC	8 (highly probable)^1^	yes	no	acetaminophen (30 g); tramadol; bromazepam; zolpidem; cetirizine	suicidal
5	M/59	HC	11 (highly probable)	yes	yes	acetaminophen (4 g/day)	abdominal pain
6	M/58	Mixed	4 (possible)	yes	no	amiodarone	atrial flutter
7	M/22	HC	7 (probable)^1^	yes	no	carbamazepine; amoxicillin/clavunilate	epilepsy; urinary tract sepsis
8	M/56	HC	10 (highly probable)^1^	yes	no	amiodarone; ibuprofen	atrial flutter; pain
9	M/63	HC	3 (possible)^1^	yes	yes	daunorubicin, cytarabine	acute leukemia, HBV reactivation
10	M/52	HC	10 (highly probable)^1^	yes	no	altiazide; bortezomib	cardiac amyloidosis
11	F/31	HC	3 (possible)^1^	yes	no	hydroxychloroquine; cyclophosphamide	lupus erythematosus
12	M/55	HC	9 (highly probable)^1^	no	no	fluvastatin; ezetimibe; fluoxetine	dyslipidemia, depression

1 When several drugs were concerned for the same patient, the RUCAM score was calculated for each drug. The RUCAM score was the same for each drug in all these cases.

HC = HepatoCellular indicates acute DILI with predominant hepatocyte necrosis; Mixed is DILI with both necrosis and cholestasis.

Three (16%) patients had DILI within less than 5 days from drug intake: chemotherapy before a bone marrow transplantation in centralized period; an overdose of acetaminophen due to abdominal pain and a suicidal case, in the standard period. In addition, 11 (58%) patients had history of drug intake more than 5 and less than 90 days.

The proportion of patients with full liver analyses, including hepatitis viral markers, auto- antibodies, and abdominal imaging was 12 out of 12 during the standard period, and 6 out of 7 during the centralized period.

After the suspicion of DILI, all patients had abdominal imaging (ultrasound or computerized tomography) to eliminate other causes of ALT increase DILI. Despite many of them have had malign diseases none of them had suspected liver images, such as nodules or liver mass.

## Discussion

There have been no studies to date that have prospectively compared a real-time strategy to a standard strategy for DILI screening in hospitals. Most studies have been retrospective and used hospital codes [4], [12]–[14]. The aim of this pilot study was to estimate the increase in DILI screening in a tertiary hospital using a proactive strategy compared with the passive standard strategy. The results of the proactive strategy were surprising: the assumed hypothesis was that the prevalence of identification would increase 6-fold, and we actually observed a 12-fold increase of DILI cases compared with the standard strategy.

The results observed during the standard period had not been underestimated. The prevalence of DILI in 6 months was 12 cases out of 30,023 total hospital admissions (0.04%) and 3.75% (12/3200) of the gastroenterology ward admissions, which were similar to those assessed retrospectively by Carey et al. (0.048% of all 83,265 hospital admissions) [13], and by Devarbhavi et al. (1.43% of 14,909 of gastroenterology ward admissions) [14]. Bargheri et al. used a computerized process based on laboratory data (ALT over 2 ULN) and noted a similar DILI rate of 6.6% in hospitalized patients [15] The authors did not perform a real-time strategy, as most of the patients were no longer hospitalized when the charts were analyzed, contrary to our strategy, in which the cases were identified in less than 48 hours.

The results from the centralized period suggest underreporting of DILI during the standard period, at least in non-hepatology wards. This was already demonstrated by Meier et al., who retrospectively observed 57 DILI cases out of 4,209 inpatients with normal baseline ALT, i.e. DILI incidence of 1.4%. Liver injuries were not mentioned in the diagnoses or in the physicians' discharge letters in about 52–68% of all cases [4].

Cases identified during the standard period were more severe than during the pro-active period, with significantly higher ALT levels, more patients who met the Hy's criteria (the conventional signal of severe DILI), and more patients who died or were transplanted. This was not a bias and was expected, since the physicians in the non-hepatology ward probably did not call the hepatologist for each case of ALT >3ULN in the absence of jaundice. These results therefore strongly suggest that the centralized method could prevent very severe complications. For instance an increase in ALT just over 3ULN in a chemotherapy patient could lead to an investigation for hepatitis B infection and to an immediate and effective treatment. This was not the case for such a patient during the standard period, who was identified only after the onset of jaundice and was treated too late and finally died from hepatic failure.

The present study had limitations due to the short period of evaluation and the commitment of eight persons. The costs and the efficiency of the centralized strategy should be confirmed over a longer period and in different hospitals to prove that the study results are generalizable (external validity). We have not evaluated a more specific strategy such as the identification of patients who have increased ALT results on two consecutive occasions. Another limitation is the possibility that treating physicians might have recognized the DILI event and thus stopped the medication or lab tests improved with time (adaption) thereby not requiring a Hepatology consult - thus over emphasizing our findings. However this bias would reduce the overall detection of DILI but not the detection of severe DILI.

There is obviously an economic interest for developing such algorithms due to the costs of ADRs in Western countries. The ADR costs in one hospital per bed and per year have been estimated to be approximately €6000 in the USA [16], France [17], and England [18]. To reduce the resources needed and particularly the physician costs, the present algorithm could be improved by using centralized hospital databases or drug event indicators [19]–[20]. Susceptibility to DILI is influenced by the interactions of many factors, including age, gender, concurrent drugs, co-morbidity and genetics. To date, no combination of factors has been able to identify very high or very low risk DILI profiles [3].

This pilot study focused on necrosis and did not detect cholestatic injury or micro/macrovesicular steatosis; the design was also not able to identify late-onset drug reactions (e.g. warfarin).

The standard of ALT >3ULN is an arbitrary guideline with low accuracy for identification of DILI [3]. Therefore it was not surprising that when the ALT guideline is used to collect possible cases, followed by clinician evaluation of the case in real-time, the accuracy of the >3ULN selection methodology then mimics the RUCAM approach or Hy's Law. We acknowledge that this strategy will only be improved with the development of more accurate DILI biomarkers (i.e. proteomics or drug-protein adduct assays), which is the aim of the SAFE-T consortium.

The implicit assumption in this study was that this real-time strategy would be more helpful than harmful to inpatients, since the offending drug would be discontinued. We recognize that it is difficult to be certain whether stopping the short-term use of life-saving medicines used in hospitals due to a possible DILI will do more good than harm [21].

On the other hand the present study shows that more DILI can be identified in real-time than in the standard “wait and see" manner. The subsequent challenge then is assessing the benefit-risk of stopping the drug. Among our identified DILI, we acknowledge that the benefit-risk of stopping amiodarone is controversial [22], but the early discovery of acute hepatitis B reactivation or olanzapine acute toxicity were probably useful for these patients.

### Conclusion

In conclusion, a simple strategy based on the daily analysis of cases identified by ALT greater than 3ULN by designated study biochemists and hepatologists identified 12 times more acute drug-induced liver disease than the standard strategy.

## Supporting Information

Checklist S1 **STROBE checklist for prospective cohort.**
(DOCX)

Protocol S1 **Protocol of the pilot study.**
(DOC)

Table S1 **Characteristics of DILI cases.**
(DOCX)

Text S1 **DILI-GHPS (Groupe Hospitalier Pitié-Salpêtrière) Group Members.**
(DOCX)
