# Supplementary material for: Real Time Identification of Drug-Induced Liver Injury (DILI) through Daily Screening of ALT Results: A Prospective Pilot Cohort Study
Source: PLoS One. 2012 Aug 14;7(8):e42418. doi: 10.1371/journal.pone.0042418 (PMC3419230; doi:10.1371/journal.pone.0042418)
Supplement: Protocol S1 — Protocol of the pilot study. (DOC) [file pone.0042418.s002.doc]

IMI SAFE-T

Clinical exploratory PoT studies and clinical biomarker assay validation

Protocol No. 3

A pilot study investigating the feasibility and requirements for a large multicenter trial on the frequency of acute drug induced liver injury (DILI) and the use of new biomarkers for DILI

| Author(s): | Thierry Poynard, Florian van Boemmel, Eckart Schott |
| --- | --- |
| Document type: | Final Protocol |
| Document status: | Final |
| Document date: | 16.03.2011 |
| Number of pages: | 18 |
|  |  |

Property of IMI SAFE-T
Confidential
May not be used, divulged, published or otherwise disclosed
without the consent of IMI SAFE-T

## Signatures

#### Authors:

Name

| Clinical Study Leader | Prof. Dr. Thierry Poynard |  | date |
| --- | --- | --- | --- |

*Name* TBD

| Study Biostatistician | Signature |  | date |
| --- | --- | --- | --- |

#### Investigator’s signature:

I have read this protocol **including the administrative and GCP related instructions contained in Part B,** and agree to conduct this trial in accordance with all stipulations of the protocol and in accordance with the Declaration of Helsinki and its amendments, and any applicable local laws and regulations.

| Prof. Dr. Thomas Berg  University Hospital of Leipzig | signature |  | date |
| --- | --- | --- | --- |
| Prof. Dr. Ulf Neumann  University Hospital of Aachen | signature |  | date |
| PD Dr. Eckart Schott  Charité, Berlin | signature |  | Date |
| Prof. Dr. Nadir Arber  Sourasky Medical Center, Tel Aviv | signature |  | date |

#### Investigators:

## Study sites:

Department of Hepato-Gastroenterology

Groupe Hospitalier Pitié Salpêtrière

47 Bd Hôpital

75651 Paris, France

Medizinische Klinik mit Schwerpunkt Hepatologie und Gastroenterologie

Charité, Campus Virchow Klinikum

Augustenburger Platz 1

D-13353 Berlin, Germany

Universitätsklinik Leipzig

Klinik und Poliklinik für Gastroenterologie und Rheumatologie

Liebigstr. 20

04103 Leipzig, Germany

Universitätsklinik Aachen, RWTH

Pauwelsstrasse 30

52074 Aachen, Germany

Tel Aviv Sourasky Medical Center

Integrated Cancer Prevention Center

3/3st Floor, Arison Medical Tower, Tel-Aviv Sourasky Medical Center

6th Weizmann St. Tel Aviv 64239, Israel

## Study title: A pilot study investigating the feasibility and the requirements for a large multicenter trial on the frequency of acute drug induced liver injury (DILI) and the use of new biomarkers for DILI

|  |  | |  |
| --- | --- | --- | --- |
| Planned dates:  first subject included: March 2011 | | last subject completed:  September 2011 | |
|  |  | |  |

**1) Background**: Many drugs can in some cases unexpectedly induce injury to human organs such as the liver, kidney or blood vessels. These kinds of side effects could be life-threatening, may lead to withdrawal of the drug treatment. Drug-induced liver injury (DILI) may occur in a dose-dependent way, as is the case for acetaminophen, or as an idiosyncratic reaction and may present as acute liver failure. Acute DILI is a rare event (14 per 100.000) but its unpredictable nature and its frequently fatal course make it a major health concern. DILI has been and still is one of the most concerning safety issues in both drug development and during the routine use of therapeutic drugs. DILI has been the most frequent single cause of safety-related drug marketing withdrawals for the past 50 years (e.g., iproniazid), continuing to the present (e.g., ticrynafen, benoxaprofen, bromfenac, troglitazone, nefazodone). Hepatotoxicity discovered after approval for marketing also has limited the use of many drugs, including isoniazid, labetalol, trovafloxacin, tolcapone, and felbamate (Temple 2001). Nowadays, there are limited possibilities to detect or monitor such organ injury. The signal currently considered most specific for and predictive of severe drug- induced liver injury is an elevation of total bilirubin levels along with clinically relevant elevations of aminotransferase activities (“Hy’s law”). However, sensitivity of this signal is inadequate to support early detection of injury as damage has already reached an extent that the function of the liver is compromised. Changes in aminotransferase activities, particularly ALT, without bilirubin elevations are more sensitive, but not sufficiently specific for drug-related liver injury. Also these current standard biomarkers are not ideal to thoroughly monitor disease progression and resolution and they do not allow to reliably predict clinical outcome of liver injury The absence of suitable detection methods complicates development of new promising medications and is a burden for many approved drugs which are already in use to treat diseases. Hence, there is a clear need for more sensitive, specific, and robust biomarkers of DILI. Better biomarkers will enable clinical decision-making in terms of safe continuation and discontinuation of drugs during clinical development as well as in routine use of marketed drugs.

As part of the European Union’s Innovative Medicines Initiative (IMI), a consortium called SAFE-T (**S**afer **A**nd **F**aster **E**vidence-based **T**ranslation) has been established in 2009 to address the urgent need for more predictive and more robust safety biomarkers for drug induced liver, kidney, and vascular injury. SAFE-T is a partnership between participants from the pharmaceutical industry, small to medium sized enterprises, academic institutions and clinical units of excellence. The consortium’s aim is to qualify promising biomarkers to detect and monitor drug-induced kidney, liver and vascular injury in humans using peripheral samples such as blood and urine. The new translational safety biomarkers will allow the identification and management of drug side effects throughout drug development, helping to reduce late stage attrition rates due to unexpected safety issues whilst at the same time improving patient safety.

In the context of SAFE-T’s DILI qualification program, the aim of this study is to collect blood samples from patients with acute DILI, and to investigate in how far a set of innovative biomarkers are associated with established DILI. In this pilot project, the feasibility of a large scale multi-center European study will be determined.

**2) Objectives:**

**2.1) Primary objective**

Main endpoint will be the time necessary to recruit 30 patients with acute DILI at four centers. This will permit to calculate the number of centers required for a large multicenter study needed for the qualification of DILI biomarker candidates as planned by SAFE-T.

**2.2) Secondary objectives**

To provide initial estimates on

1. Relationships between ALT and the new biomarkers proposed by the SAFE-T consortium in DILI diagnosis

b) Prevalence of necro-inflammatory DILI versus others different types of acute DILI such as cholestatic and steatotic DILI

c) Percentage of patients lost to post hospitalization follow-up

1. Type of drugs suspected in DILI
2. Prevalence of associated liver risk factors such as alcohol consumption, metabolic factors, history of familial liver disease, history of drug adverse events, history of recreational drugs (cocaine, ecstasy, amphetamines) and herbals.

**3) Patients and Methods**

**3.1) Patients**

A total of 30 patients with acute DILI will be included. Fifteen patients will be recruited in Paris and 15 patients will be recruited at the three German sites (Berlin, Aachen, Leipzig). The duration of follow-up per patient will be 3 months. An estimated 500 patients will have to be included in the multicenter core study that will follow-up on this pilot study.

**3.1.1) Criteria for inclusion**

Consecutive patients with suspected acute DILI as defined by:

1. ALT activitiy exceeding 3 x ULN or ALP > 2 x ULN, with baseline activities before drug treatment, if available, < ULN.
2. history of drug intake including history of intake of acetaminophen, multiple drugs, recreational drugs (cocaine, ecstasy, amphetamines), herbals
3. for baseline ALT or ALP activity, if available, > ULN, an increase of at least 2-fold the baseline level
4. absence of known other causes of liver injury
5. patients with age > 18 years that are capable of and willing to provide written informed consent

**Specific considerations:**

- Patients with Amanita phalloides intoxication will be included but analyzed separately
- Patients with acute HBV flares due to immuno-suppressive drugs/chemotherapy will be included but analyzed separately
- Patients with metabolic syndrome (obesity, diabetes, known NAFLD, known NASH) or excessive alcohol consumption (male > 40 g/day, female > 20 g/day) will be included but analyzed separately
- HIV infected patients will be included if an acute DILI is suspected

**3.1.2) Criteria for exclusion:**

a) Chronic hepatitis C

b) Autoimmune liver disease

c) Primary biliary cirrhosis (PBC)

d) Primary sclerosing cholangitis (PSC)

e) Extrahepatic cholestasis

f) Ischemic liver damage

g) Presence of liver metastasis of other malignant diseases

**3.2) Study Design**

A multicenter, prospective, observational, non-interventional study in patients with suspected acute DILI

**3.3) Methods**

Proper risk assessment and signal detection as early as possible in clinical drug development are crucial to identify any potential of a drug to cause idiosyncratic liver toxicity. However, even with drugs having a well established potential to cause idiosyncratic hepatocellular injury, incidences of severe DILI can be as low as 1 in 10,000 patients treated. Patients fulfilling Hy’s law criteria have a risk of around 10% to develop severe liver injury, so instead of true, clinical injury, Hy’s law cases may be a suitable indicator of a drug’s potential to cause relevant liver problems and hence could be used to qualify new liver safety biomarkers. However, even that would require sample sizes of some ten thousand patients to accumulate a sufficient number of Hy’s cases for robust statistical characterization of any marker. Given the time frame of five years for the SAFE-T consortium to achieve adequate qualification of selected liver biomarkers, it is highly unlikely that sample sizes required will be obtained when using Hy’s law only as signal definition. A signal considered reasonably sensitive next to Hy’s law are cases exceeding 3 x ULN for ALT without associated elevation of bilirubin, which has also been coined as “Temple’s corollary”. The consortium will therefore focus on cases fulfilling Temple’s corollary (ALT exceeding 3 x ULN) [Temple R, 2006] rather than Hy’s law only to define cases suitable for biomarker qualification.

For cholestatic liver injury cases, cut-off level for alkaline phosphatase will be 2 x ULN.

*Causality assessment:* Cases with suspected DILI should be initially ascertained by clinical judgement of the physician in charge and, subsequently, by the evaluation of three experts based on the following criteria: 1) an appropriate temporal relationship between the intake of the drug and the onset of the event, 2) the improvement of liver damage following the withdrawal of the drug, 3) exclusion of other causes of liver disease, 4) relapse following re-exposure when applicable, and 5) previous reports of the adverse reaction. Cases will be further evaluated for causality assessment, by application of the Council for International Organizations of Medical Science (CIOMS)/Roussel Uclaf Causality Assessment Method (RUCAM) scale.

**3.3.1) DILI Biomarkers**

The SAFE-T DILI team performed a careful and exhaustive review of the current literature and in house data from partners. Approximately 45 potential biomarker candidates were evaluated, using a standard set of criteria. Of the 45 initially evaluated biomarker candidates, 22 were recommended for clinical qualification. The core set of biomarker candidates to be assessed within the consortium comprises: micro RNA 122, albumin mRNA, high mobility group box protein 1 (HMGB-1), ALT 1 & 2 isozymes, F-protein (HPPD), arginase 1, cytokeratin 18, microglobulin precursor (Ambp) mRNA, alpha-fetoprotein, regucalcin, urocanic acid, glutathione S-transferase (GST-alpha), alpha 2,6-sialytransferase (ST6gal), osteopontin, colony stimulating factor receptor (CSF1R), glutamate dehydrogenase (GLDH), purine nucleoside phosphorylase (PNP), malate dehydrogenase (MDH), conjugated/unconjugated bile acids, paraoxonase 1 (PON1)/prothrombin and Leucocyte cell-derived chemotaxin2 (LECT2).

In addition to the DILI biomarker candidates listed above, liver disease markers used for clinical diagnosis will be qualified for detection of DILI. The tests that will be used are:

**Fibrotest™, ActiTest**™ **, SteatoTest**™ and **FibroScan**™

**FibroTest**™ is a patented non-invasive biomarker test that is reported to have the same diagnostic value as a biopsy. It is a multi-parametric test that utilizes a combination of 6 blood serum tests to generate a fibrosis score that is correlated with the degree of liver damage in patients with a variety of liver disease. This test is proposed, associated with **ActiTest**™, for use as an alternative to liver biopsy for the assessment of fibrosis stage in the four more common chronic liver diseases: hepatitis C virus, hepatitis B virus, hepatitis nonalcoholic fatty liver disease (NAFLD), and alcoholic liver disease (ALD).

**ActiTest**™ is a biomarker of liver necro- inflammatory histological activity initially validated

in patients with chronic hepatitis C and B

**SteatoTest**™ is a biomarker of liver steatosis validated in patients with non-alcoholic steatosis, alcoholic steatosis, and chronic hepatitis C.

**3.3.2) Time-points of evaluation and samples management**

Patients will be evaluated and will have blood sample collection at baseline, day 1, day 3, week 1, week 2, week 3, week 4, week 8 and week 12. Baseline will be defined as the day of first visit. The sample volume required for measuring the biomarkers is 24 ml of blood at each time-point: 10ml of blood in a dry tube, 5 ml in EDTA tube, 5ml in Li-Heparin tube and 4ml tube containing hexokinase inhibitor. The total amount of blood drawn will be 216 ml in three months in addition to routine tests. The potential risk of sample collection is phlebitis. This can be a local inflammation or in very rare cases, it can lead to a systemic infection. In addition 15 ml of urine will be sampled.

**3.3.2.1) Data collection**

Medical and personal data will be collected at each time-point. All these data will be recorded within the framework of the SAFE-T consortium and entered into a personal file in the test centre and saved electronically. All medical and personal data collected will be kept under strict confidentiality at the responsibility of the SAFE-T consortium. The patient’s name will never be attached to those data (completely anonymous).

a) The following data will be collected at baseline:

- Inclusion/exclusion criteria
- Diagnosis of concomitant diseases
- Screen for HAV (anti-HAV), HBV (HBs-Ag, anti-HBc), HCV (anti-HCV), HIV (anti-HIV)
- Demography, smoking history
- Liver ultrasound
- Alcohol test (Carboxy Deficient Transferin), drug screen and pregnancy test

b) The following data will be collected at each time-point (including baseline):

- Current medical conditions (symptomatology, date of onset of symptoms, interval between start of medication use and liver dysfunction)
- Current medication in use (date of start and dosage of current medications. Use of recreational drugs and herbals)
- Physical examination including body weight, height, body mass-index and vital signs
- Complete blood count
- Coagulation profile (PTT, INR, TPT)
- Clinical Chemistry (ALT, AST, GGT, LDH, total, direct/indirect bilirubin, alkaline phosphatase, total protein, albumin, BUN/urea, creatinine, ferritin, transferrin, uric acid, cholesterol, triglycerides, glucose, HbA1C, calcium, magnesium, phosphorus, sodium, potassium, chloride, bicarbonate, amylase and lipase, creatine kinase (CK), CK-MB)
- Urine Analysis (Quantitative spot urine for protein, microalbumin, and creatinine Urinalysis consisting of color &clarity, specific gravity, pH, protein, glucose, ketones, bilirubin, urobilinogen, blood, nitrite, leukocyte esterase, Urine microscopic WBC, WBC)
- Thyroid Function (TSH)
- Previous FibroScan and FibroTest/ActiTest data, if available

**3.3.2.2) Management of the blood samples**

After sample’s collection, all of them will be centrifuged, the serum or plasma will be extracted and they will be stored frozen (-80°) at the study site. All biological samples will be coded. The patient’s name will never be attached to those samples (completely anonymous). The frozen samples will be regularly sent for a centralized biobank at Barcelona (SAFE-T Biobank) under responsibility of the SAFE-T consortium. At the SAFE-T Biobank the samples will be aliquoted in 300 microliters tubes and the biomarkers will be measured.

**4) Ethical considerations**

This study will be conducted in accordance with the protocol and in accordance with :

- The declaration d’Helsinki of 1964.

- The international rules and regulations governing GCP (1996)

- The charter on ethical research in developing countries edited by the ANRS (May 2002)

**4.1) Informed consent**

The patients will be informed in a transparent and complete fashion, in layman’s terms, of the objectives and constraints of the study, of the potential risks associated with the study, of the monitoring, of the patient’s right to decide not to participate in the study and of the patient’s right to withdraw from the study at any time in the future. All this information will be contained in the patient information sheet in the same document as the informed consent form. A written informed consent by the patients will be collected by the investigator, or a medical representative of the investigator, before recruitment into the study. A copy of the patient information sheet and of the signed informed consent form, signed by both the patient and the investigator, or the investigator’s representative, will be given to the patient, the investigator keeping one of the original copies. At the end of the study, one copy of this information sheet and consent form will be placed in a sealed envelope including all the other signed informed consent forms of the patients recruited into the study, and this envelope will be archived.

**4.2) Access to study data and source documents**

Patients’ data collected during the study will be kept strictly confidential and anonymous. For audit purposes, only the medical and scientific representatives, involved in the conduct of the study, in addition to a representative person from the health authority will be allowed to access the patients’ medical source data.

**4.3) Quality control and Quality assurance**

In accordance with Good clinical practice (GCP), and in order to guarantee the quality of the study and protect the patients recruited in the study,each investigator of the study will commit to

- The regular monitoring and audit of the study.

- An eventual audit of the study, conducted by relevant organisations

- An audit of the study by the health authorities.

Guidance and monitoring plans will be written and communicated to the investigators. A CRF will be assigned to each patient recruited in the study, with a study number and the code linking this study number to the identification of the patient.

**4.4) Retention of the documents and data related to the study**

The investigators in the study will archive the documents related to the study, and will keep these documents available for any potential audit for at least 15 years.

Such documents related to the study are as follows:

- updated version of the protocols, appendices, with any amendments.

- CRF

- Informed consent signed by the patients and the confidential list of patients having taking part in the study, linking each patient to the study number and the patient’s source hospital case notes.

- Correspondence related to the study

The source data file must be available for a period of 15 years.

**5) Statistical methods**

The **primary endpoint** will be the time necessary to recruit 30 patients with acute DILI at four centers. The number of patients eligible per month per center will permit to calculate the number of centers needed for a large multicenter study including 500 cases for the qualification of DILI biomarker candidates as planned by SAFE-T.

**5.1) Statistical Methods for Estimating Patient Accrual Rates**

For Estimating Patient Accrual Rates, the time intervals between the recruitment of successive patients will be collated. With the assumption that the inter-arrival times of patients are stochastically independent, the times will be assumed to have come from an exponential distribution and the (single) parameter will be estimated. This will allow probabilistic statements to be made concerning the likely time required to recruit the desired number of patients in future studies.

This procedure will be carried out on the pooled data from all centres in the study. Exploratory analyses will allow for the mean inter-arrival times to vary between the centres, by estimating the parameters separately and by fitting the parameters to a gamma distribution. This last step will allow some prediction of the time to recruit required sample sizes, allowing for likely variability in recruitment rates between centres.

Several **secondary endpoints** are defined for this study.

Initial estimates will be given for

- relationship between ALT and the new biomarkers proposed by the SAFE-T consortium for DILI diagnosis,
- the prevalence of necro-inflammatory DILI versus other different types of acute DILI such as cholestatic and steatotic DILI ,
- the percentage of patients lost to post hospitalization follow-up,
- the prevalence of associated liver risk factors such as alcohol consumption, metabolic factors, history of familial liver disease, history of drug adverse events, history of recreational drugs (cocaine, ecstasy, amphetamines), and use of herbals.

An initial overview of the type of drugs suspected in DILI will be obtained.

The nature of the protocol is exploratory and there are many unknowns. Therefore, some of the predefined analyses described below will likely be modified to some degree in the light of the data. Also, further analyses may be performed as questions raised by the data present themselves.

The relationship between ALT and the new biomarkers will be estimated in an exploratory way using graphical presentation where appropriate. Special attention will given to extreme ALT values.

The prevalence of necro-inflammatory DILI versus other different types of acute DILI such as cholestatic and steatotic DILI, the prevalence of associated liver risk factors such as alcohol consumption, metabolic factors, history of familial liver disease, history of drug adverse events, history of recreational drugs (cocaine, ecstasy, amphetamines), and use of herbals, and the estimated percentage of patients lost to post hospitalization follow-up will be evaluated using graphical presentations and summary statistics by center and in total.

**5.2) Statistical Methods for Exploring the Distribution and Relationships Between Biomarkers**

All biomarkers will be summarized by their minimum, quartiles, and maximum at each scheduled clinic visit, including absolute values, absolute changes from baseline and proportional changes from baseline. Boxplots and shiftplots will be presented on a log scale for each biomarker at each clinic visit. For any patient of particular interest, time profiles of relevant lab variables will be plotted.

Correlation between new biomarkers and ALT (at each scheduled clinic visit, and maximum absolute and proportional change from baseline) will be summarized using a robust estimate of correlation, specifically using an S-estimator, following Maronna et al (2006, page 204). Dependence between the changes from baseline in the extremes will be summarized using the χ and χ-bar statistics described by Coles et al (1999), plotted together with approximate 90% confidence intervals. Also, the conditional multivariate Spearman’s ρ estimate of Schmid and Schmidt (2007) will be presented and a 90% confidence interval will be estimated by the bootstrap.

Scatterplots of each biomarker versus each other biomarker, with lines joined in time order, will be presented and non linear mixed effect models may be applied in order to aid understanding of the time course of relationships between biomarkers.

For multivariate modelling, lasso and gradient boosted models will be used (Hastie et al, 2010). If the response variable is binomial (e.g. presence/absence of liver injury), lasso logistic regression and gradient boosted logistic regression will be used.

# Appendix 1: ALT ULNs by study site

| **Center** | **Males** | **Females** |
| --- | --- | --- |
| Charité Berlin | 45 IU/L | 34 IU/L |
| University Hospital of Aachen | 51 U/L | 35U/L |
| APHP Paris | 35 IU/L | 26 IU/L |
| University Hospital of Leipzig | 0.85 μkat/L | 0.60 μkat/L |
| TASMC Tel Aviv | 35 ?? | ?? |
