# Supplementary material for: Real Time Identification of Drug-Induced Liver Injury (DILI) through Daily Screening of ALT Results: A Prospective Pilot Cohort Study
Source: PLoS One. 2012 Aug 14;7(8):e42418. doi: 10.1371/journal.pone.0042418 (PMC3419230; doi:10.1371/journal.pone.0042418)
Supplement: Table S1 — Characteristics of DILI cases. (DOCX) [file pone.0042418.s003.docx]

**Supplementary Table S1**

|  | **Potential DILI drugs started together ?** | **Time between drug start and increased ALT (days)** | **Medication only during hospitalisation** | **use of heparin** | **TB > 2xULN** | **TB max**  **(mmol/L)** | **PT < 50%** | **PT min**  **(ref 70-120%)** | **use of AVK** |
| --- | --- | --- | --- | --- | --- | --- | --- | --- | --- |
| **Centralized period** |  |  |  |  |  |  |  |  |  |
| 1 | yes | 10 | yes | no | no | 8 | no | 94 | no |
| 2 | yes | 5 | yes | no | yes | 39 | no | 103 | no |
| 3 | yes | 17 | yes | no | no | 8 | no | 118 | no |
| 4 | yes | 15 | no | no | yes | 41 | no | 95 | no |
| 5 | yes | 76 | no | no | no | 8 | no | 107 | no |
| 6 | yes | 2 | yes | no | no | 14 | no | 114 | no |
| 7 | yes | 14 | yes | yes (LMWH) | no | 24 | no | 71 | no |
| **Standard period** |  |  |  |  |  |  |  |  |  |
| 1 | yes | 157 | no | no | yes | 586 | yes | 16 | no |
| 2 | yes | 40 | yes | yes (LMWH) | no | 7 | no | 98 | no |
|  |  |  |  |  |  |  |  |  |  |
| 3 | yes | 2 | no | no | yes | 352 | yes | 14 | no |
| 4 | yes | 2 | no | no | no | 32 | yes | 17 | no |
| 5 | yes | 52 | no | no | yes | 306 | yes | 11 | no |
| 6 | yes | 136 | no | no | no | 26 | yes | 37 | yes - atrial flutter |
| 7 | no | carbamazepine - 326  amox / clav - 45 | no | no | yes | 281 | yes | 48 | no |
| 8 | no | amiodarone - 10 ibuprofen - 15 | no | no | no | 19 | yes | 27 | yes - atrial flutter |
| 9 | yes | 240 | no | no | yes | 439 | yes | 32 | no |
| 10 | yes | 67 | no | no | no | 21 | no | 100 | no |
| 11 | yes | 174 | no | no | no | 20 | no | 94 | no |
|  |  |  |  |  |  |  |  |  |  |
| 12 | yes | 42 | no | no | no | 15 | NA | NA | no |

DILI: drug induced liver injury; ALT: alanine transaminase; LMWH: low-molecular weight heparin; TB: total bilirubin; PT: prothrombin time; ULN: upper limit of normal; AVK: anti-vitamin K
