# Supplementary material for: Real Time Identification of Drug-Induced Liver Injury (DILI) through Daily Screening of ALT Results: A Prospective Pilot Cohort Study
Source: PLoS One. 2012 Aug 14;7(8):e42418. doi: 10.1371/journal.pone.0042418 (PMC3419230; doi:10.1371/journal.pone.0042418)
Supplement: Text S1 — DILI-GHPS (Groupe Hospitalier Pitié-Salpêtrière) Group Members. (DOCX) [file pone.0042418.s004.docx]

**Supporting information file Text S1**

***Drug Induced Liver Injury (DILI) Groupe Hospitalier Pitié-Salpêtrière (GHPS): DILI-GHPS Group Members:***

**Administration:** Vincent Nicolas Delpech

**Metabolic Biochemistry:** Dominique Bonnefont-Rousselot, Françoise Imbert-Bismut, Helmi Mkada.

**Biochemistry Unit (Emergency):** Catherine Devillier

**Cardiology:** Komajda Michel

**Consultation Medicine:** Christian De Gennes

**Diabetology:** Agnes Hartemann

**Endocrinology Metabolism:** Eric Bruckert

**Endocrinology Reproduction:** Philippe Touraine

**Geriatry:** Marc Verny

**Gynecology Obstetrics:** Marc Dommergues

**Hematology:** Veronique Leblond, Jean Paul Vernant

**Hepato-Gastroenterology:** Thierry Poynard, Vlad Ratziu, Dominique Thabut, Jean Baptiste Bachet, Marika Rudler, Julien Massard.

**Infectious Disease:** Bricaire Francois, Christine Katlama, Martin Danis

**Inflammatory Disease:** Catherine Lubetzki

**Information Technology:** Philippe Boulogne, Jean-claude Merrien, Pierre Rufat

**Intensive Care Anesthesiology:** Pierre Coriat

**Internal Medicine:** Zahir Amoura, Herson Serge

**Medical Intensive Care:** Jean Chastre, Alexandre Duguet

**Emergency Medicine:** Bruno Riou

**Nuclear Medicine:** Andre Aurengo

**Nutrition:** Arnaud Basdevant, Karine Clément

**Parasitology:** Mazier Dominique

**Pulmonology:** Thomas Similowski

**Neonatology:** Frederique Quetin

**Neurology Stroke Center:** Yves Samson

**Neurology 1:** Charles Pierrot Deseilligny

**Neurology 2:** Jean Yves Delattre

**Neurology 3:** Michel Baulac

**Neurology 4:** Olivier Lyon Caen Bruno Dubois, David Cohen, Vincent Meininger

**Nephrology:** Gilbert Deray

**Oncology:** David Khayat

**Ophthalmology:** Phuc Le Hoang

**Pharmacology:** Patrick Tilleul, Amélie Liou

**Psychiatry:** Roland Jouvent, Allilaire Jean Francois

**Radiation Therapy:** Jean Jacques Mazeron

**Rheumatology:** Bruno Fautrel, Pierre Bourgeois, Cécile Gaujoux-Viala

**Stomatology:** Patrick Goudot

**Surgery Cardiac:** Alain Pavie

**Surgery, Endocrinology:** Fabrice Menegaux

**Surgery, Hepatobiliary:** Hannoun Laurent, Jean Christophe Vaillant

**Surgery, Gynecology:** **Jean-Pierre Lefranc**

**Surgery, Neurology:** Philippe Cornu

**Surgery, Orthopedic:** Yves Catonne

**Surgery, Urology** Marc Olivier Bitker, François Richard

**Surgery, Vascular:** Fabien Koskas
